# Myoglobin clearance with continuous veno-venous hemodialysis using high cutoff dialyzer versus continuous veno-venous hemodiafiltration using high-flux dialyzer: a prospective randomized controlled trial

**DOI:** 10.1186/s13054-020-03366-8

**Published:** 2020-11-11

**Authors:** Lorenz Weidhase, Jonathan de Fallois, Elena Haußig, Thorsten Kaiser, Meinhard Mende, Sirak Petros

**Affiliations:** 1 Medical Intensive Care Unit, University Hospital Leipzig, Leipzig, Saxony Germany; 2 Division of Nephrology, Medical Department III, University Hospital Leipzig, Leipzig, Saxony Germany; 3 Institute for Laboratory Medicine, Clinical Chemistry and Molecular Diagnostics, University Hospital Leipzig, Leipzig, Saxony Germany; 4 Institute for Medical Informatics, Statistics and Epidemiology, University Leipzig, Leipzig, Saxony Germany

**Keywords:** Myoglobin clearance, Rhabdomyolysis, Acute kidney injury, High cutoff dialyzer, Renal replacement therapy, EMiC2

## Abstract

**Background:**

Myoglobin clearance in acute kidney injury requiring renal replacement therapy is important because myoglobin has direct renal toxic effects. Clinical data comparing different modalities of renal replacement therapy addressing myoglobin clearance are limited. This study aimed to compare two renal replacement modalities regarding myoglobin clearance.

**Methods:**

In this prospective, randomized, single-blinded, single-center trial, 70 critically ill patients requiring renal replacement therapy were randomized 1:1 into an intervention arm using continuous veno-venous hemodialysis with high cutoff dialyzer and a control arm using continuous veno-venous hemodiafiltration postdilution with high-flux dialyzer. Regional citrate anticoagulation was used in both groups to maintain the extracorporeal circuit. The concentrations of myoglobin, urea, creatinine, β2-microglobulin, interleukin-6 and albumin were measured before and after the dialyzer at 1 h, 6 h, 12 h, 24 h and 48 h after initiating continuous renal replacement therapy.

**Results:**

Thirty-three patients were allocated to the control arm (CVVHDF with high-flux dialyzer) and 35 patients to the intervention arm (CVVHD with high cutoff dialyzer). Myoglobin clearance, as a primary endpoint, was significantly better in the intervention arm than in the control arm throughout the whole study period. The clearance values for urea and creatinine were higher in the control arm. There was no measurable albumin clearance in both arms. The clearance data for β_2_-microglobulin and interleukin-6 were non-inferior in the intervention arm compared to those for the control arm. Dialyzer lifespan was 57.0 [38.0, 72.0] hours in the control arm and 70.0 [56.75, 72.0] hours in the intervention arm (*p* = 0.029).

**Conclusions:**

Myoglobin clearance using continuous veno-venous hemodialysis with high cutoff dialyzer and regional citrate anticoagulation is better than that with continuous veno-venous hemodiafiltration with regional citrate anticoagulation.

**Trial registration:**

German Clinical Trials Registry (DRKS00012407); date of registration 23/05/2017. https://www.drks.de/drks_web/navigate.do?navigationId=trial.HTML&TRIAL_ID=DRKS00012407.

## Background

Acute kidney injury (AKI) is one of the leading organ dysfunctions in critically ill patients. According to the multinational acute kidney injury–epidemiologic prospective investigation (AKI-EPI) study, more than half of critical care patients suffer from AKI and this is associated with high mortality rates, particularly if renal replacement therapy (RRT) is required [1–3].

Several pathogenetic mechanisms are involved in the development of AKI in critically ill patients. Hemodynamic alterations or inflammation-related stress may trigger renal damage [4].

One precisely defined cause of AKI is rhabdomyolysis, characterized by damage of skeletal muscles and the leakage of muscle cell contents into the circulation, e.g., myoglobin and other proteins [5]. Up to 7–10% of AKI is attributed to rhabdomyolysis [6]. The reported incidence of AKI in patients with rhabdomyolysis is 13–46% [7], and RRT may be necessary in these patients.

Continuous renal replacement therapy (CRRT) may be favorable in these critical ill patients, since it enables gentle removal of solutes and control of fluid balance. However, it is not yet clear if continuous or intermittent hemodialysis would be the best regarding clinical outcome [8–10].

Current international guidelines recommend anticoagulation with citrate for CRRT, unless systemic anticoagulation is required for other indications or citrate use is contraindicated [11]. Regional citrate anticoagulation (RCA) is associated with less bleeding complications, longer lifespan of the dialyzer and lower incidence of heparin-induced thrombocytopenia (HIT) than systemic anticoagulation [12, 13].

Convection-based RRT techniques such as continuous veno-venous hemofiltration (CVVH) require higher extracorporeal circuit blood flow than diffusion-based ones, which is related to hemoconcentration at the dialyzer. Therefore, RCA during CVVH is hardly possible due to the high risk of citrate accumulation.

Continuous veno-venous hemodialysis (CVVHD) enables reducing citrate load [14], so that this mode using RCA is favorable in critically ill patients. One disadvantage of diffusive techniques is the poor clearance of molecules with middle molecular weight [15]. Continuous veno-venous hemodiafiltration (CVVHDF) is one opportunity to realize a low blood flow with consecutive lower citrate load and maintain middle molecule clearance.

Another solution for this problem could be the application of high cutoff (HCO) membranes with a pore size larger than 0.01 μm in CVVHD (CVVHD-HCO) [16]. It has already been shown that CVVHD using a high cutoff dialyzer is superior than that using a conventional high-flux dialyzer regarding the clearance of β_2_-microglobulin, a molecule with a middle molecular weight [17]. The application of HCO dialyzers in CVVHDF is not recommended, because it leads to albumin leakage, particularly with postdilution hemodiafiltration [18, 19].

Based on this finding, we have designed a prospective, randomized controlled trial to evaluate the myoglobin clearance with CVVHDF compared to that with CVVHD-HCO using RCA. Clearance of myoglobin is important due to its direct toxic effects. Clinical data comparing different modalities of CRRT addressing myoglobin clearance are lacking.

## Methods

### Study design

The present study is a prospective, randomized, single-blinded, single-center trial. It was approved by the local ethics committee, conducted in accordance with the German medical product law and registered at the German Clinical Trials Registry (DRKS00012407).

We enrolled patients from May 2017 to September 2018 in our 28-bed medical intensive care unit (ICU) at the University Hospital Leipzig. Informed consent was either given by the patients or their legal guardians. Allocation concealment and unrestricted randomization was carried out using sequentially numbered, opaque sealed envelopes as previously described [20]. In detail, a sheet of standard-sized paper marked with the treatment arm was folded to fit an envelope, with 35 each marked for treatment arms A and B. To prevent attempts to decipher the allocation sequence, one sheet of carbon paper was put on top of this paper and wrapped with aluminum foil. This was then inserted in an opaque envelope and sealed. The 70 sealed envelopes were then thoroughly mixed and marked with unique numbers, and then kept in a container [20]. For technical reasons, only patients were blinded to the treatment arm.

### Patients

During the study period, 430 patients with acute renal failure and indication for RRT (based on the recommendations of the kidney disease, improving global outcomes (KDIGO)) [11] were admitted to the ICU and were screened for eligibility. Exclusion criteria were need for systemic anticoagulation for other reasons, high risk for citrate accumulation (e.g., liver failure), pregnancy and lactation, age < 18 years, rejection of renal replacement therapy, refusal to participate in the study, high risk to die during the first 48 h after admission and end-stage underlying disease.

We planned to show non-inferiority in myoglobin clearance as the primary end-point in the intervention arm compared to the control arm. Based on previous trials, a difference in myoglobin clearance of not more than 1.85 ml/min between the two treatment arms was considered to show non-inferiority of the intervention arm [17, 21]. Based on that, the inclusion of 66 patients was calculated to be required with a two-tailed power of 90% and *p* of < 0.05. Our study protocol allowed testing for superiority after demonstration of non-inferiority of the intervention arm [22].

**Fig. 1:**
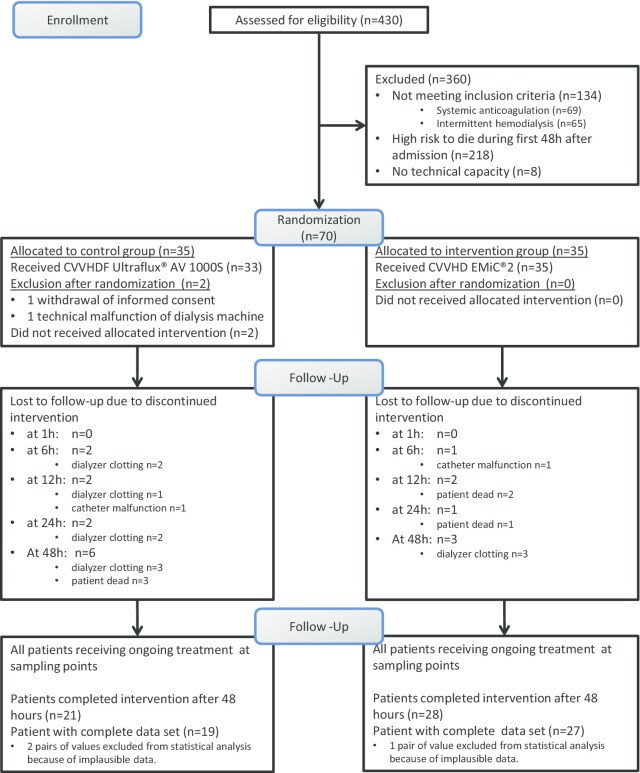
Recruitment flowchart. *CVVHDF* continuous veno-venous hemodiafiltration, *CVVHD* continuous veno-venous hemodialysis, *AV1000S* type of high-flux dialyzer, *EMiC2* type of high cut-off dialyzer

A total of 70 patients underwent randomization 1:1 into the intervention and control arms. We later excluded two patients, one due to withdrawal of informed consent and another patient because of technical malfunction of the dialysis machine. Three pairs of values were excluded from statistical analysis because of implausible data, presumably related to preanalytical errors. Recruitment flowchart is displayed in Fig. 1.

### Data collection

Demographic and clinical data were collected in all patients. Baseline criteria were recorded at time of initiating RRT (renal function, admission diagnosis, indication for renal replacement therapy, acute physiology and chronic health evaluation II (APACHE II), sequential organ failure assessment (SOFA), simplified acute physiology score II (SAPS II), mean arterial blood pressure, need for mechanical ventilation, need for vasopressor, sepsis, concomitant medication and pre-existing diseases). Laboratory and clinical data during the intervention were daily collected (urea, creatinine, sodium, potassium, chloride, phosphate, calcium, magnesium, hemoglobin, hematocrit, platelet count, white blood cell count, albumin, pH, bicarbonate, base excess, lactate, mean arterial pressure, heart rate and oxygen saturation).

Clinical follow-up data (ICU mortality, length of ICU stay, 28-day mortality and 90-day mortality) were extracted from patient records.

Treatment-related adverse events (AE) (hypocalcemia, alkalosis, citrate accumulation, catheter malfunction) and severe adverse events (SAE) (treatment associated life-threatening complication and death from any cause during intervention period) were monitored.

### Procedure

A central venous access using a 13 French double-lumen high-flow catheter (Achim Schulz-Lauterbach VMP, Iserlohn, Germany) was placed. The control arm was managed with CVVHDF postdilution using the high-flux dialyzer Ultraflux AV1000S (Fresenius Medical Care, Bad Homburg, Germany), while the intervention arm was managed with CVVHD using the high cutoff dialyzer Ultraflux EMiC2 (Fresenius Medical Care, Bad Homburg, Germany). Both dialyzers have the same effective surface area (1.8 m^2^), consist of identical material (polysulfone) and exhibit a similar wall thickness (35 µm). The only difference between the applicated dialyzers is the pore size, which is 30 kilodaltons (kDa) for the high-flux and 45 kDa for the high cutoff dialyzer.

The dialysis machine used in both arms was multiFiltrate® (Fresenius Medical Care, Bad Homburg, Germany). A bicarbonate-buffered dialysate (CiCa® dialysate K4 or K2, Fresenius medical care, Bad Homburg, Germany) and replacement fluid (multiBic® K4 or K2, Fresenius medical care, Bad Homburg, Germany) were used. Anticoagulation of the extracorporeal circuit was maintained with regional citrate anticoagulation in both arms. Regional anticoagulation of the extracorporeal circuit was monitored by measuring ionized postfilter calcium and guided by citrate supply (citrate: 136 mmol/l). An ionized postfilter calcium of 0.20–0.29 mmol/l for CVVHDF and 0.25–0.34 mmol/l for CVVHD was targeted. At the start of treatment, citrate flow was set at 5.0 mmol citrate/l blood for CVVHDF and 4.0 mmol citrate/l blood for CVVHD. To keep systemic ionized calcium stable between 1.12 and 1.20 mmol/l, a calcium chloride solution (calcium: 83 mmol/l) was added to the extracorporeal circuit near the backflow to the patient. Both groups started with a flow of 1.7 mmol Ca^2+^/l dialysate.

The total turnover rate (TTR) (dialysate and replacement fluid) rate was calculated at 25 ml/kg ideal or adjusted body weight/h [23]. Ideal body weight was calculated using the Hamwi equation (for males: 48 kg for the first 152 cm + 1.1 kg for each additional cm; for females 45 kg for the first 152 cm + 0.9 kg for each additional cm). If the quotient of actual body weight divided by ideal body weight was more than 1.3 [24], the adjusted body weight was used for calculation of dialysate flow (for males: (actual body weight-ideal body weight) * 0.38 + ideal body weight; for females: (actual body weight-ideal body weight) * 0.32 + ideal body weight) [25].

The ratio of dialysate flow to replacement fluid in CVVHDF was 2:1, and blood flow (QB) was threefold of the dialysate flow in both arms as specified by the manufacturer. According to the statement of the manufacturer, the maximum dialyzer lifespan was limited to 72 h.

### Endpoints and calculations

The concentrations of myoglobin (17,053 Dalton (Da), urea (60 Da), creatinine (113 Da), β2-microglobulin (11,800 Da), interleukin 6 (IL-6, 26,000 Da) and albumin (66,470 Da) were measured before (*C*_pre_) and after (*C*_post_) the dialyzer 1, 6, 12, 24 and 48 h after initiating CRRT.

To avoid additional effect of hemoconcentration at the dialyzer, ultrafiltration was set at zero ten minutes before drawing samples for laboratory analysis.

Plasma flow in the extracorporeal circuit (Qp_pre_) was calculated using blood flow (Qb) of extracorporeal circuit and patient's hematocrit level (hct) at the time of sampling:

$$\text{Qp}_\text{pre}\,\text{(ml/min)}=\text{Qb}\times ((1-\text{hct})\div 100)$$

The substance-specific plasma clearance (Cl_p_) was calculated at the sampling time points:

$$\text{Cl}_\text{p }\,(\text{ml/min})=\text{Qp}_\text{pre}\times (({C}_\text{pre}-{C}_\text{post.})\div {C}_\text{pre})$$

The primary outcome parameter was plasma clearance of myoglobin after 1 h, 6 h, 12 h, 24 h and 48 h. Secondary outcome parameters were plasma clearances of urea, creatinine, β2-microglobulin, interleukin 6 and albumin at the same time points.

Equality of plasma clearance (Cl_p.corr._) in both study groups relating to different TTR was tested using the following formula:

$$\text{Cl}_\text{p.corr.}=\text{Cl}_\text{p}\times \left(\frac{\text{TTR}_{\text{CVVHD-HCO}}}{{\text{TTR}}_{\text{CVVHDF}}}\right)$$

In CVVHDF (control arm), a sampling port was available only before instead of after the replacement fluid flows into the extracorporeal circuit. Therefore, the postfilter solute concentration (*C*_post_) had to be corrected to account for replacement fluid flow.

For this purpose, postfilter plasma flow (Qp_post_) was calculated subtracting filtration portion (FP; ml/min) from prefilter plasma flow (Qp_pre_):

$$\text{Qp}_\text{post}\,(\text{ml/min})=\text{Qp}_\text{pre}-\text{FP}$$

The solute-specific concentration at the end of the extracorporeal circuit (*C*_post.corr._) was adjusted using the ratio of plasma flow pre- and postfilter (Qp_post_/Qp_pre_)_:_

$${C}_\text{post.corr.}\,(\text{mmol/l})={(\text{Qp}}_{\text{post}}\div {\text{QP}}_{\text{pre}})\times {C}_{\text{post}}$$

### Laboratory analysis

Laboratory analyses were performed using Cobas 8000 (Roche, Mannheim, Germany) according to the manufacturer´s instructions, immediately after sampling.

The following methods were used:

- Urea: kinetic test with urease and glutamate dehydrogenase.
- Creatinine: enzymatic method with creatinase.
- β2-microglobulin: am c701 immunological test for turbidity.
- Myoglobin: ElektroChemiLumineszenzImmunoAssay (ECLIA).
- IL-6: ECLIA.
- Human albumin: color test with bromocresol green.

### Statistical analysis

We planned the trial as a non-inferiority trial keeping in mind to present the results in terms of superiority if the effect is large enough. Sample size was calculated cautiously for a non-inferiority study. We assumed a mean of 11.1 for the intervention arm and 7.4 for the control arm, with a standard deviation of 6.75 for myoglobin clearance from pilot data and a significance level *α* = 5%. The non-inferiority margin was chosen a sixth of standard deviation, which is 1.85 ml/min. The study was planned to show non-inferiority in values of myoglobin clearance with 90% power. A sample size of 66 was calculated using the software PASS 2008 for non-inferiority. Taking 5% dropouts into account, this results in a sample size *N* = 70. The study was not powered to show differences in the other substance specific clearances.

Continuous variables are given as mean with standard deviation or median with 25th and 75th quantile in square brackets based on test for normal distribution using the Shapiro–Wilk test. Categorical variables are displayed as n (%). Normally distributed variables were analyzed by the Student’s *t* test. Not normally distributed variables were assessed by the Mann–Whitney *U* test. Categorical variables were tested by Chi-square (two-sided), reported as frequencies and percentages. Measures of clearance are repeated measurements from the same patients and thus are correlated. Therefore, we fitted general linear models with repeated measurements. Differences between the arms were calculated at each time point with 95% confidence interval (CI). Marginal means were compared and the difference was calculated with 95% CI as effect measure. A test of non-inferiority was performed with these confidence intervals.

The Kaplan–Meier method was applied to calculate and depict the survival function of the dialyzer lifetime.

Statistical analysis was performed using IBM SPSS, versions 24 to 26 (Minneapolis, USA). R (R Core Team, Vienna) was applied for the generation of graphs. The significance level was defined 5% for two-tailed tests.

## Results

### Baseline criteria

Serum creatinine concentration was significantly higher in the control arm. There were no differences regarding other baseline variables between both study arms (Table 1).

**Table 1:** Baseline characteristics of the study population

Variable	Control arm CVVHDF, *n* = 33	Intervention arm CVVHD-HCO, *n* = 35	*p* value
Age, years	67 [51,74.5]	68 [57,74.0]	0.626
Males	26 (78.8%)	18 (51.4%)	0.180
Actual body weight, kg	80 [72,100]	80 [70,90]	0.341
Height, cm	174.70 ± 10.58	170.83 ± 9.16	0.113
BMI	26 [24, 30]	27 [24, 30]	0.873
APACHE II score	29.27 ± 7.27	29.60 ± 8.11	0.861
SOFA score	9.15 ± 3.57	10.09 ± 3.88	0.305
SAPS II score	56.27 ± 14.29	57.43 ± 14.63	0.743
Creatinine (µmol/l)	240 [170,413]	188 [129,266]	0.031
Urea (mmol/l)	19.90 ± 8.80	19.14 ± 8.99	0.728
urine output during first 24 h (ml)	530 ± 752	518 ± 602	0.941
Admission diagnosis			
Sepsis	9 (27.3%)	11 (31.4%)	
Pneumonia	3 (9.1%)	3 (8.6%)	
ARDS	3 (9.1%)	4 (11.4%)	
AKI	2 (6.1%)	3 (8.6%)	
Hemorrhage	4 (12.1%)	1 (2.9%)	
Cardiac	4 (12.1%)	5 (14.3%)	
Rhabdomyolysis	2 (6.1%)	0 (0%)	
Pancreatitis	1 (3.0%)	2 (5.7%)	
Liver failure	1 (3.0%)	5 (14.3%)	
Other	4 (12.1%)	1 (2.9%)	
Systolic BP, mmHg	108.33 ± 20.94	108.29 ± 20,04	0.992
MAP mmHg	70 [62,78.5]	72 [63,80]	0.961
Mechanical ventilation	24 (72.7%)	33 (94.3%)	0.16
Vasopressor	23 (69.7%)	32 (91.4%)	0.23
Pre-existing diseases			
Congestive heart failure NYHA IV	6 (18.2%)	8 (22.9%)	0.634
Pre-existing immunosuppression	9 (27.3%)	17 (48.6%)	0.71
Liver cirrhosis	7 (21.2%)	10 (28.6%)	0.484
History of malignancy	7 (21.2%)	11 (31.4%)	0.34
Chronic pulmonary disease	3 (9.1%)	8 (22.9%)	0.123
Indication for RRT			
Metabolic acidosis	5 (15.2%)	12 (34.3%)	0.069
Pulmonary edema	12 (36.4%)	15 (42.9%)	0.584
Hyperpotassemia	8 (24.2%)	7 (20%)	0.673
Anuria (< 100 ml/d)	26 (78.8%)	25 (71.4%)	0.484
Uremia	20 (60%)	13 (37.1%)	0.53
Main reason for AKI			
Septic	13 (39.4%)	19 (54.3%)	
Postrenal	0 (0%)	0 (0%)	
Cardiorenal	4 (12.1%)	5 (14.3%)	
Toxic	3 (9.1%)	0 (0%)	
Hypovolemia	6 (18.2%)	3 (8.6%)	
Rhabdomyolysis	3 (9.1%)	0 (0%)	
Hepatorenal	2 (6.1%)	5 (14.3%)	
Other	2 (6.1%)	3 (8.6%)	

Data presented as *n* (%), mean ± standard deviation or median [25th, 75th quantile]

*BMI* body mass index, *APACHE II* acute physiology and chronic health evaluation II, *SOFA* sequential organ failure assessment, *SAPS II* simplified acute physiology score II, *ARDS* Acute Respiratory Distress Syndrome, *AKI* acute kidney injury, *BP* blood pressure, *MAP* mean arterial blood pressure, *RRT* renal replacement therapy, *CVVHDF* continuous veno-venous hemodiafiltration, *CVVHD-HCO* continuous veno-venous hemodialysis using high cutoff filter

Blood flow effectively obtained in both treatment arms was equal (after 1 h: control arm: 86.4 ± 23 ml/min; intervention arm: 91.7 ± 18 ml/min; *p* = 0.3). TTR in the control arm was higher (after 1 h: control arm: 2116 ± 328 ml/h; intervention arm: 1821 ± 351 ml/h; *p* = 0.001). Data are displayed in an additional file (see Additional file 1).

### Primary endpoint

**Fig. 2:**
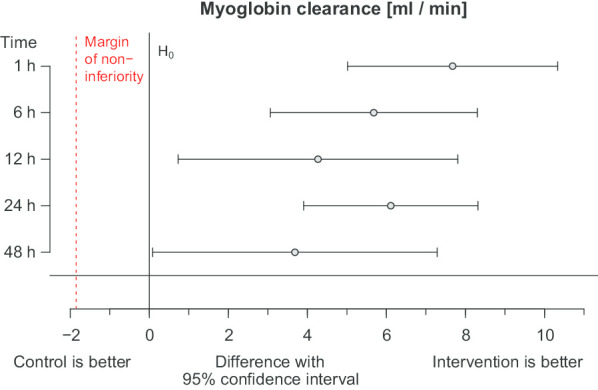
Non-inferiority of myoglobin clearance. The mean arm differences with 95% confidence intervals at 1, 6, 12, 24, 48 h. The null hypothesis *Δ* = 0 (no arm difference) was tested against the alternative hypothesis *Δ* > −1.85 ml/min (margin of non inferiority), depicted by the dashed red line

**Fig. 3:**
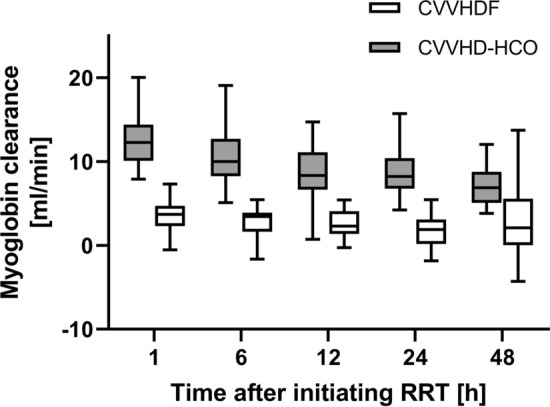
Myoglobin clearance at different time points. Graphical analysis in grouped boxplots. *ml/min* milliliters per minute, *RRT* renal replacement therapy, *CVVHDF* continuous veno-venous hemodiafiltration, *CVVHD* continuous veno-venous hemodialysis

The 95% confidence intervals for the difference of myoglobin clearance were at the pre-specified margin − 1.85 for non-inferiority at all time points. Indeed, these confidence intervals are right from zero indicating a significant advantage of the intervention versus control arm (Fig. 2). This is reflected by a significant comparison of the marginal means (*p* < 0.0005, Table 2) too. Myoglobin clearance was higher in the intervention arm than in the control arm at every time point (Fig. 3).

**Table 2:** Substance specific clearances (ml/min) (primary and secondary endpoints)

End-points	Time after starting treatment	*n*	Control arm CVVHDF^†^	Intervention arm CVVHD-HCO^†^	Mean difference^‡^	*p* value^#^
Myoglobin	1 h	66	3.7 [2.3, 4.7]	12.3 [10.1, 14.4]	5.5 (4.0–7.0)	< 0.0005
	6 h	65	3.4 [1.6, 3.9]	10 [8.3, 12.7]		
	12 h	62	2.3 [1.5, 4]	8.3 [6.7, 10.7]		
	24 h	57	1.9 [0.2, 3.1]	8.2 [6.8, 10.3]		
	48 h	48	2.1 [0.1, 5.5]	6.9 [5.1, 8.8]		
Urea	1 h	66	28.7 [25.6, 33.2]	24.8 [20.9, 28.7]	− 5.3 (− 8.4− (− 2.3))	0.001
	6 h	65	29.6 [25, 31.4]	25.1 [22.8, 28.3]		
	12 h	62	28.8 [25.2, 32.9]	26.1 [19.8, 28.8]		
	24 h	57	28.6 [25.8, 32.4]	26.7 [20.9, 29.7]		
	48 h	48	31 [26.2, 32.8]	23 [20.4, 30.9]		
Creatinine	1 h	66	31.4 [27.6, 35.9]	28.5 [24.7, 34.1]	4.2 (0.8–7.6)	0.015
	6 h	65	31.7 [27.2, 34.5]	29.5 [25.3, 34.9]		
	12 h	62	31.1 [27.8, 34.8]	29.5 [22.5, 33]		
	24 h	58	31.5 [28, 37.1]	30.4 [24.6, 35.9]		
	48 h	48	32,9 [27.5, 36.5]	25.7 [23.7, 34.7]		
β2− Microglobulin	1 h	66	21.1 [18, 23.8]	24.5 [19.2, 28.1]	4.4 (2.2–6.6)	< 0.0005
	6 h	65	19 [17.1, 22.4]	24.1 [21,1, 26.6]		
	12 h	62	7.8 [4.5, 10.9]	22.7 [18.2, 24.5]		
	24 h	57	16.4 [15.5, 19.4]	22.1 [19.2, 25.8]		
	48 h	48	18 [15.5, 20.4]	21.4 [16.9, 23.7]		
Interleukin− 6	1 h	66	0.2 [− 0.9, 1.2]	5 [2.4, 5.9]	1.4 (− 0.3–3.1)	0.107
	6 h	65	0.3 [− 1, 2.5]	2.5 [1, 3.5]		
	12 h	62	1.2 [0, 3.7]	1.8 [0.6, 3.4]		
	24 h	57	− 0.6 [− 1.3, 1.5]	1.6 [0.4, 3]		
	48 h	48	0.7 [− 1.1, 4.2]	1.3 [0, 2.3]		
Albumin	1 h	66	− 3.4 [− 4.2, − 1.1]	− 1.2 [− 2.6, 0.3]	− 1.1 (− 2.7–0.5)	0,170
	6 h	65	− 2.1 [− 3.5, 0.3]	− 1.4 [− 3, − 0.2]		
	12 h	62	− 1.4 [− 2.9, 1.7]	− 1.7 [− 3.1, − 0.1]		
	24 h	57	− 2.3 [− 4.5, − 0.8]	− 1.6 [− 3.2, − 07]		
	48 h	48	− 1.7 [− 5.7, 1.2]	− 2.4 [− 5, − 0.8]		

Data presented as † median [25th, 75th quantile], ‡ Estimated difference of marginal means (95% confidence interval)

*ml/min* milliliters per minute, *CVVHDF* continuous veno-venous hemodiafiltration, *CVVHD-HCO* continuous veno-venous hemodialysis using high cutoff filter

^#^*p* value from comparison of marginal means

The mean arm differences *Δ* with 95% confidence intervals at 1, 6 12, 24 and 48 h. The null hypothesis *Δ* = 0 (no arm difference) was tested against the alternative hypothesis *Δ* > − 1.85 ml/min (margin of non-inferiority), depicted by the dashed red line.

Graphical analysis in grouped boxplots. *ml/min* milliliters per minute, *RRT* renal replacement therapy, *CVVHDF* continuous veno-venous hemodiafiltration, *CVVHD* continuous veno-venous hemodialysis,

### Secondary endpoints

The clearance values for urea and creatinine were higher in the control arm than in the intervention arm. After accounting for the higher TTR in the control arm, clearance values were equal in both arms (Cl_p.corr._) and displayed in an additional file (see Additional file 2).

The clearance for β_2_-microglobulin was better in intervention group. Although no significant difference in IL-6 and albumin clearance could be observed concerning marginal means, IL-6-clearance was higher after 1, 6 and 24 h and albumin clearance after 1 h in the intervention arm (see Additional file 3).

### Dialyzer lifespan

**Fig. 4:**
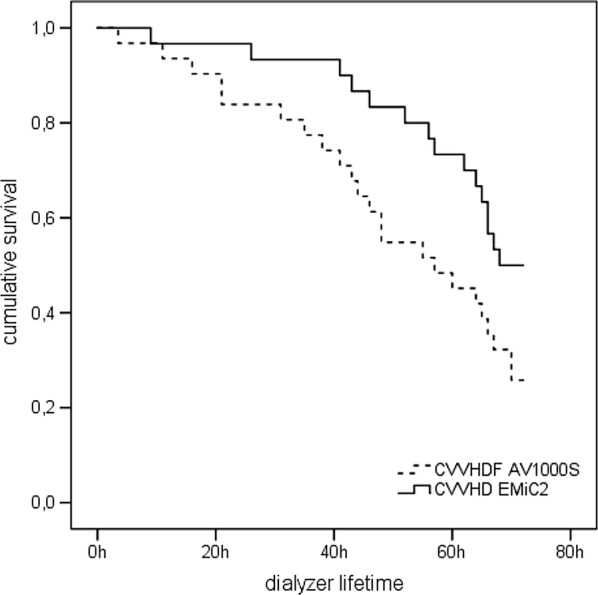
Kaplan–Meier plot for dialyzer lifespan. *CVVHDF* continuous veno-venous hemodiafiltration, *CVVHD* continuous veno-venous hemodialysis, *AV1000S* type of high-flux dialyzer, *EMiC2* type of high cut-off dialyzer

Dialyzer lifespan was reported as Kaplan–Meier plot. Six patients were excluded from this analysis because treatment was terminated prematurely for other reasons. Median dialyzer lifespan was 57.0 [38.0, 72.0] hours in the control arm and 70.0 [56.75, 72.0] hours in the intervention arm. This difference was statistically significant (log-rank (Mantel-Cox), *p* = 0.029, Fig. 4).

### Safety endpoints

ICU mortality was higher in the intervention arm than in the control arm. There were no differences in hospital, 28- and 90-day mortality. Adverse events and severe adverse events did not differ between the groups (Table 3).

**Table 3:** Safety endpoints

Variable	Control arm CVVHDF, *n* = 33	Intervention arm CVVHD-HCO, *n* = 35	*p* value
Clinical outcome			
ICU length of stay (days)	11 (5.21)	13 (7.27)	0.543
ICU mortality	10 (30.3%)	22 (62.9%)	0.007
Hospital mortality	18 (54.5%)	24 (68.6%)	0.234
Mortality day 28	15 (45.5%)	21 (60%)	0.23
Mortality day 90	21 (65.6%)	25 (73.5%)	0.485
Adverse events			0.205
Hypocalcemia	1 (3%)	6 (17.1%)	
Metabolic alkalosis	1 (3%)	0 (0%)	
Citrate accumulation	0 (0%)	0 (0%)	
Catheter malfunction	1 (3.3%)	1 (2.9%)	
Severe adverse events			0.265
Treatment-associated life-threatening complication	0 (0%)	0 (0%)	
Dead of any cause during intervention	2 (6.1%)	5 (14.3%)	

Data presented as *n* (%). median [25th, 75th quantile]

*ICU* intensive care unit, *CVVHDF* continuous veno-venous hemodiafiltration, *CVVHD-HCO* continuous veno-venous hemodialysis using high cutoff filter

## Discussion

The present study demonstrated that plasma myoglobin clearance in patients with AKI requiring RRT is significantly higher using CVVHD with a high cutoff dialyzer compared to that of CVVHDF with a high-flux dialyzer.

In contrast to recent clinical trials which evaluated the clearance of β_2_-microglobulin with HCO membranes [17, 26–29], we investigated myoglobin as primary endpoint. High myoglobin levels are associated with AKI and higher mortality rates [6, 7]. Therefore, myoglobin clearance represents a more relevant clinical issue in AKI than the clearance of β_2_-microglobulin. Serum myoglobin is the best parameter to indicate rhabdomyolysis and can provide the best prediction for AKI in these cases [30, 31]. One retrospective analysis showed that 68.9% of elderly people with rhabdomyolysis developed AKI [32].

Fast elimination of myoglobin is essential due to its direct toxic renal effects [33]. Myoglobin is endocytosed by tubular cells and oxidized, resulting in radical oxygen species that alter DNA and protein function. It activates an inflammatory response in the kidney and mediates vasoconstriction, which perpetuate renal damage. Myoglobin is also filtered by the glomerulus and precipitates in the renal tubules, particularly in combination with the Tamm–Horsfall proteins, forming tubular casts, which consequently result in acute tubular obstruction [5, 33].

Because working kidneys are able to remove more myoglobin than any extracorporeal system [34], the first therapeutic choice is prevention of myoglobinuric AKI and maintain diuresis with adequate fluid resuscitation [35, 36]. RRT might be necessary in patients in whom fluid resuscitation cannot prevent AKI. Clinical data addressing extracorporeal removal of myoglobin are lacking, and it is not yet clear if that prevents AKI, influences the duration of oliguria/anuria or reduces mortality.

Hemofiltration is recommended to eliminate myoglobin in patients with myoglobinuric AKI who need RRT [21]. Due to the hemoconcentration along the dialyzer, CVVH requires higher blood flow than CVVHD. Thus, CVVH with RCA and the required higher blood flow would imply higher citrate load. Systemic anticoagulation increases the risk of bleeding complications, particularly after traumatic rhabdomyolysis, and it may also be associated with HIT [12, 13]. Therefore, CVVHD with RCA using HCO dialyzer could be one possible solution to this problem. Another possibility to reduce citrate load and ensure myoglobin clearance is the combination of hemofiltration and hemodialysis (CVVHDF).

Effective elimination of myoglobin by CVVHDF in rhabdomyolysis was first reported by Mikkelsen and Toft [37]. Albert et al. showed a decline of myoglobin levels using HCO dialyzer [38]. This result was in accordance with another case series involving 18 patients with rhabdomyolysis who underwent longer treatment (6–10 h) with HCO hemodialysis [39]. The effectiveness of HCO dialyzers to eliminate myoglobin was confirmed in another case series with sustained low efficiency daily dialysis (SLEDD) and CVVHD [40]. The cutoff values for the HCO dialyzers used in these studies were similar to the HCO dialyzer in our study, although the effective membrane surface area differed [38–40].

A good clearance of β_2_-microglobulin was demonstrated in chronic dialysis patients using dialyzers with increased pore size [26, 27]. Recent studies have also confirmed a good clearance for middle molecules in CVVHD with RCA and HCO dialyzers in critically ill patients [17, 28].

Better clearance of β_2_-microglobulin in the intervention arm in this study is in line with the results of others [17, 28, 41]. Contrary to these findings, another small cohort (*n* = 10), which compared the β_2_-microglobulin clearance between CVVHDF using high-flux and HCO dialyzers, showed no difference in both study arms, although the dialysis dose was much higher in the CVVHDF arm (36 ± 4 ml/kg/h) than in the CVVHD arm (21 ± 6 ml/kg/h) [29].

High levels of β2-microglobulin were observed in patients with end-stage renal disease and might be presumed to be a prognostic parameter in chronic dialysis patients [42, 43]. However, the prognostic role of β2-microglobulin in acute kidney injury and critical care medicine and whether if effective clearance of this molecule could be associated with survival advantage is not elucidated.

Initial albumin loss during CVVH using HCO dialyzers was reported in an older study [19]. That is why we used the HCO dialyzers only in CVVHD. With the exception of the sample after 1 h we did not observe differences in albumin clearance in both study groups, which is similar to the findings of recent studies [16, 17, 41].

HCO dialyzers show a greater clearance for inflammatory cytokines than conventional high-flux membranes [16, 44, 45]. Interleukin-6 clearance in our study was superior in the intervention arm after 1, 6 and 24 h, but showed no difference at the remaining time points compared to the control arm. Possible reasons could be the higher molecular weight of Il-6 compared to that of myoglobin and the so-called membrane fouling by protein adsorption and polarization within the membranes over time [16]. This observation was in accordance with the findings of another study on 30 patients comparing HCO and high-flux dialyzers in CVVHD with RCA [46]. In that study, the HCO arm showed a higher clearance of IL-6 and interleukin 10 and decreasing clearance values during the study period [46]. Recent findings strengthen the hypothesis that the clearance for molecules with higher molecular weight decreases over time according to the specific molecular weight [28]. Experimental data support the results of lower clearance rates at a molecular weight greater than 30 kDa and the time dependent influence on middle molecule clearance [47]. The higher albumin clearance after 1 h in CVVHD-HCO group supports this assumption.

Higher TTR had to be realized in the control arm to maintain calcium homeostasis. This accounted for higher clearance values for urea and creatinine in this group. However, no clearance differences of these two small molecules could be detected after correcting for TTR. We therefore conclude that CVVHD using HCO dialyzers is not inferior than CVVHDF using high-flux dialyzers regarding urea and creatinine clearance.

The observed shorter dialyzer lifespan in our control arm in comparison with our intervention arm may be related to hemoconcentration at the dialyzer during CVVHDF, which may contribute to clot formation [48]. This effect could be enhanced by the postdilution modality. Another reason might be the more error-prone management of CVVHDF with RCA, related to different dialysis and replacement fluids. Older studies comparing CVVHDF with RCA versus systemic anticoagulation with heparin showed no advantage regarding dialyzer survival using RCA [49, 50]. This seems to be a specific problem using CVVHDF with RCA. Contrary to these older studies, newer studies, although using CVVHDF, showed longer dialyzer survival using RCA similar to the findings of other trials using other modes of CRRT with RCA [12, 13].

The high observed mortality in both treatment arms is in line with results of other studies considering the disease severity of investigated patient population [51].

### Limitations

There are certain limitations to our trial. Firstly, it is a monocentric, single-blinded trial, conducted at a medical ICU. A double-blind design was impossible, because the extracorporeal circuit differs between the two study arms.

Secondly, we analyzed the performance of two different renal replacement methods concerning solute specific clearances in critical care patients who suffered AKI for different reasons. It is not possible to conclude that the choice of procedure effects mortality or renal outcome. Thirdly, we cannot provide any recommendation when RRT should be started to remove myoglobin. Finally, the primary endpoint consists of 5 repeated measurements. However, we believe that using the marginal mean for comparison of the arms is a good choice, which also addresses the problem of multiple tests sufficiently.

## Conclusion

In summary, this study demonstrates that myoglobin can be cleared using CVVHD-HCO with regional citrate anticoagulation in AKI in critical ill patients in a medical ICU.

This study provides a solid background to generate hypotheses and design large clinical trials with hard clinical end points. Further studies are needed to show non-inferiority of CVVHD-HCO compared to CVVH with systemic anticoagulation, especially in cases of severe rhabdomyolysis.

In our opinion, CVVHD using high cutoff dialyzers with RCA could be beneficial in patients suffering from AKI and high myoglobin values, particularly in conditions with high risk of bleeding.

## Supplementary information

**Additional file 1**. Dialysis protocol.

**Additional file 2**. Substance specific clearances (ml/min) after 1h and adjustment for TTR.

**Additional file 3**. Substance specific clearances (ml/min) at different time points.

## Data Availability

The datasets used and analyzed during the current study are available from the corresponding author on reasonable request.

## Abbreviations

AKI: Acute kidney injury
RRT: Renal replacement therapy
CRRT: Continuous renal replacement therapy
RCA: Regional citrate anticoagulation
HIT: Heparin-induced thrombocytopenia
CVVH: Continuous veno-venous hemofiltration
CVVHD: Continuous veno-venous hemodialysis
CVVHDF: Continuous veno-venous hemodiafiltration
HCO: High cutoff
KDIGO: Kidney disease, improving global outcomes
ICU: Intensive care unit
kDa: Kilodaltons
TTR: Total turnover rate (dialysate and substituent)
QB: Blood flow
Il-6: Interleukin-6
Cpre: Concentration before the dialyzer
Cpost: Concentration after the dialyzer
Cpost.corr: Corrected concentration after the dialyzer
Qppre: Prefilter plasma flow
Hct: Hematocrit level
Clp: Substance-specific plasma clearance
Clp.corr.: Corrected plasma clearance
Qppost: Postfilter plasma flow
FP: Filtration portion
ECLIA: ElektroChemiLumineszenzImmunoAssay
CI: Confidence interval
SLEDD: Sustained low efficiency daily dialysis
