# Supplementary material for: Myoglobin clearance with continuous veno-venous hemodialysis using high cutoff dialyzer versus continuous veno-venous hemodiafiltration using high-flux dialyzer: a prospective randomized controlled trial
Source: Crit Care. 2020 Nov 11;24:644. doi: 10.1186/s13054-020-03366-8 (PMC7659077; doi:10.1186/s13054-020-03366-8)
Supplement: Supplementary file 1 — Additional file 1. Dialysis protocol. [file 13054_2020_3366_MOESM1_ESM.pdf]

Additional file 1: Dialysis protocol

Variable	*Calculated BF*	*Calculated TTR*	*BF T0 (1h)*	*BF T1 (6h)*	*BF T2 (12h)*	*BF T3 (24h)*	*BF T4 (48h)*	*TTR T0 (1h)*	*TTR T1 (6h)*	*TTR T2 (12h)*	*TTR T3 (24h)*	*TTR T4 (48h)*
CVVHDF	69.36±14.06	2039.98±398.56	86.36±23	88.06±23	88±22	87.41±19	88.1±21	2116±328	2101±315	2137±327	2133±302	2111±334
CVVHD-HCO	91.18±19.19	1814.76±379.47	91.71±18	91.76±18	93.44±18	93.55±18	90.69±16	1821±351	1803±396	1895±346	1879±370	1816±355
P value	<0.05	0.020	0.300	0.485	0.301	0.229	0.643	0.001	0.002	0.008	0.008	0.010

Data presented as mean ± standard deviation. *BF* blood flow (ml/min), *TTR* total turnover rate (ml/h), *CVVHDF* continuous veno-venous hemodiafiltration, *CVVHD-HCO* continuous veno-venous hemodialysis using high cut-off filter.
