# Supplementary material for: Myoglobin clearance with continuous veno-venous hemodialysis using high cutoff dialyzer versus continuous veno-venous hemodiafiltration using high-flux dialyzer: a prospective randomized controlled trial
Source: Crit Care. 2020 Nov 11;24:644. doi: 10.1186/s13054-020-03366-8 (PMC7659077; doi:10.1186/s13054-020-03366-8)
Supplement: Supplementary file 2 — Additional file 2. Substance specific clearances (ml/min) after 1h and adjustment for TTR. [file 13054_2020_3366_MOESM2_ESM.pdf]

Additional file 2: Substance specific clearances (ml/min) after adjustment for TTR:

End-points	***$Cl_{p.corr.}$ Control group (CVVHDF)***	***Cl_p Intervention group (CVVHD-HCO)***	***P value***	***n***
Urea	24.67 (22.08,28.60)	24.81 (20.93,28.72	0.995	66
Creatinin	27.04 (23.77,30.9)	28.51 (24.72,34.14)	0.272	66
β2-Microglobulin	18.13 (15.46,20.49)	24.48 (19.22,28.13)	<0.001	66
Myoglobin	3.18 (1.98,4.06)	12.27 (10.07,14.41)	<0.001	66
Interleukin-6	0.19 (-0.75,1.02)	4.96 (2.39,5.88)	<0.001	66
Albumin	-2.92 (-3.61,-0.95)	-1.24 (-2.57,0.25)	0.046	66

Data presented as median (25th, 75th quantile). *ml/min* milliliters per minute, *CVVHDF* continuous veno-venous hemodiafiltration, *TTR* total turnover rate, *CVVHD-HCO* continuous veno-venous hemodialysis using high cut-off filter, Cl_p plasma clearance, $Cl_{p.corr.}$ corrected plasma clearance
