# Supplementary material for: Myoglobin clearance with continuous veno-venous hemodialysis using high cutoff dialyzer versus continuous veno-venous hemodiafiltration using high-flux dialyzer: a prospective randomized controlled trial
Source: Crit Care. 2020 Nov 11;24:644. doi: 10.1186/s13054-020-03366-8 (PMC7659077; doi:10.1186/s13054-020-03366-8)
Supplement: Supplementary file 3 — Additional file 3. Substance specific clearances (ml/min) at different time points. [file 13054_2020_3366_MOESM3_ESM.pdf]

Additional file 3: Substance specific clearances (ml/min) at different time points

End-points	*Time after starting treatment*	*n*	*Control arm* CVVHDF	*Intervention arm* CVVHD-HCO	*P value*
Myoglobin	**1h**	66	3.7 [2.3, 4.7]	12.3 [10.1, 14.4]	<0.001
	**6h**	65	3.4 [1.6, 3.9]	10 [8.3, 12.7]	<0.001
	**12h**	62	2.3 [1.5, 4]	8.3 [6.7, 10.7]	<0.001
	**24h**	57	1.9 [0.2, 3.1]	8.2 [6.8, 10.3]	<0.001
	**48h**	48	2.1 [0.1, 5.5]	6.9 [5.1, 8.8]	<0.001
Urea	**1h**	66	28.7 [25.6, 33.2]	24.8 [20.9, 28.7]	0.005
	**6h**	65	29.6 [25, 31.4]	25.1 [22.8, 28.3]	0.025
	**12h**	62	28.8 [25.2, 32.9]	26.1 [19.8, 28.8]	0.008
	**24h**	57	28.6 [25.8, 32.4]	26.7 [20.9, 29.7]	0.106
	**48h**	48	31 [26.2, 32.8]	23 [20.4, 30.9]	0.031
Creatinin	**1h**	66	31.4 [27.6, 35.9]	28.5 [24.7, 34.1]	0.094
	**6h**	65	31.7 [27.2, 34.5]	29.5 [25.3, 34.9]	0.227
	**12h**	62	31.1 [27.8, 34.8]	29.5 [22.5, 33]	0.111
	**24h**	58	31.5 [28, 37.1]	30.4 [24.6, 35.9]	0.299
	**48h**	48	32,9 [27.5, 36.5]	25.7 [23.7, 34.7]	0.083
β2-Microglobulin	**1h**	66	21.1 [18, 23.8]	24.5 [19.2, 28.1]	0.053
	**6h**	65	19 [17.1, 22.4]	24.1 [21,1, 26.6]	<0.001
	**12h**	62	7.8 [4.5, 10.9]	22.7 [18.2, 24.5]	<0.001
	**24h**	57	16.4 [15.5, 19.4]	22.1 [19.2, 25.8]	<0.001
	**48h**	48	18 [15.5, 20.4]	21.4 [16.9, 23.7]	0.063
Interleukin-6	**1h**	66	0.2 [-0.9, 1.2]	5 [2.4, 5.9]	<0.001
	**6h**	65	0.3 [-1, 2.5]	2.5 [1, 3.5]	0.007
	**12h**	62	1.2 [0, 3.7]	1.8 [0.6, 3.4]	0.573
	**24h**	57	-0.6 [-1.3, 1.5]	1.6 [0.4, 3]	0.009
	**48h**	48	0.7 [-1.1, 4.2]	1.3 [0, 2.3]	0.640
Albumin	**1h**	66	-3.4 [-4.2, -1.1]	-1.2 [-2.6, 0.3]	0.020
	**6h**	65	-2.1 [-3.5, 0.3]	-1.4 [-3, -0.2]	0.511
	**12h**	62	-1.4 [-2.9, 1.7]	-1.7 [-3.1, -0.1]	0.799
	**24h**	57	-2.3 [-4.5, -0.8]	-1.6 [-3.2, -07]	0.443
	**48h**	48	-1.7 [-5.7, 1.2]	-2.4 [-5, -0.8]	0.424

Data presented as Median [25th,75th quantile]. *ml/min* milliliters per minute, *CVVHDF* continuous veno-venous hemodiafiltration, *CVVHD-HCO* continuous veno-venous hemodialysis using high cut-off filter
